# A 2.09 Mb fragment translocation on chromosome 6 causes abnormalities during meiosis and leads to less seed watermelon

**DOI:** 10.1038/s41438-021-00687-9

**Published:** 2021-12-01

**Authors:** Shujuan Tian, Jie Ge, Gongli Ai, Jiao Jiang, Qiyan Liu, Xiner Chen, Man Liu, Jianqiang Yang, Xian Zhang, Li Yuan

**Affiliations:** State Key Laboratory of Crop Stress Biology for Arid Areas, College of Horticulture, Northwest A&F University, Yangling, 712100 Shaanxi China

**Keywords:** Developmental biology, Experimental organisms

## Abstract

Seedlessness is a valuable agronomic trait in watermelon (*Citrullus lanatus*) breeding. Conventional less seed watermelons are mainly triploid, which has many disadvantages due to unbalanced genome content. Less seed watermelon can be achieved at the diploid level when certain reproductive genes are mutated or by chromosome translocation, which leads to defects during meiosis. However, the formation mechanism of diploid less seed watermelons remains largely unknown. Here, we identified a spontaneous mutant line, watermelon line “148”, which can set seeds normally when self-pollinated. A total of 148 × JM F_1_ hybrid plants exhibited seed number reductions to 50.3% and 47.3% of those of the two parental lines, respectively, which are considered to be less seed. Examination of pollen viability and hybridization experiments revealed that F_1_ hybrids produce semisterile pollen and ovules. Further cytological observations indicated that semisterility was a result of a reciprocal translocation of chromosomes, which exhibited one quadrivalent ring of four chromosomes at prometaphase I during meiosis. RT-qPCR analysis indirectly confirmed that the semisterile phenotype is caused by chromosome translocation rather than disruption of specific meiotic gene expression. F_2_ population genetic analysis indicated that the “148” watermelon line is a homozygous translocation and that the less seed phenotype of the F_1_ hybrid is prompted by one chromosome fragment translocation. The translocated fragment was further fine mapped to a 2.09 Mb region on chromosome 6 by whole-genome resequencing and genetic map cloning procedures. Our work revealed that a 2.09 Mb chromosome fragment translocation on chromosome 6, causing meiotic defects at metaphase I during meiosis, leads to diploid less seed watermelon. Our findings provide a new promising method for less seed watermelon breeding at the diploid level, as well as a fragment size reference for breeding less seed watermelon through artificially induced chromosome translocation.

## Introduction

Less seed fruits are highly valuable and desirable agronomic traits in economically important fruit crop breeding. Due to their easy consumption, the demand for less seed fruit varieties is increasing throughout the world. In addition, less seed fruits have many gustatory advantages. For example, studies have shown that less seed tomato fruits are tastier than seeded varieties, exceed seeded fruits in dry matter content by up to 1%^1^, and contain more sugars, less acidity, less cellulose, and considerably more soluble solids than seeded cultivars^2^. Research on less seed traits for plant breeding has been increasingly awarded to molecular and biotechnological breeding. However, only a few less seed fruit mutants have been characterized in agronomic and horticultural economic crops and are able to be used in crop breeding. In tomato, the *CHS* gene is involved in flavonoid biosynthesis, and the silencing of *CHS* leads to less seed tomato fruits^3^. In addition, *SlHAK5* contributes to pollen K^+^ uptake and viability, and *slhak5* knockout (KO) lines produce almost less seed fruits^4^. In pear, *PbGA20ox2*, a GA 20-oxidase gene, is highly expressed in young fruits and plays key roles in less seed pear fruit development by enhancing GA content^5^. Whether less seed horticultural economical crop fruits can be achieved by alternative breeding programs needs to be further characterized.

Watermelon is an economically important horticultural crop and one of the top five most consumed fresh fruits worldwide (http://faostat.fao.org). One fruit of watermelon commonly contains several hundred seeds. Therefore, less seed watermelon fruit is a desirable trait and of great value for consumers due to its lack of seeds, consumer preference, better fruit quality, and greater economic value to growers than traditional seeded cultivars^6,7^. The less seed fruits are mainly categorized into two types: parthenocarpy and stenospermocarpy. Parthenocarpy, in which the fruit develops in the absence of fertilization, has been identified in cultivated pineapples, some citrus cultivars, and bananas. The less seed watermelon production methods of the parthenocarpy type mainly include the application of plant growth regulators (i.e., cytokinin) to pistillate flowers^8^ and to pollinating seeded pistillate flowers with sterile pollen grains from seeded cultivators irradiated by X or gamma rays^9–11^. Stenospermocarpy, where pollination and fertilization are required, embryos either do not form seeds or abort before completion of seed formation^12,13^. Less seed watermelon contains partially developed seeds and is a classic example of stenospermocarpy. Two main mechanisms or pathways are responsible for the formation of less seed watermelon fruits. One method is that a traditional cross is made between a tetraploid maternal parent and a diploid pollinator, resulting in a triploid plant that is self-infertile because of a gametic chromosome imbalance. This triploid plant is pollinated by a diploid plant to produce less seed watermelon^14–16^. Nevertheless, there are some disadvantages of the above traditional less seed watermelon fruit technology^17^: 1) a tedious production process of F_1_ seeds (commercial triploid seeds); 2) a low yield and relatively high cost of F_1_ seeds; 3) difficulty in the germination of F_1_ seeds or of nursing their seedings; and 4) late maturity. To overcome these undesirable characteristics, a second less seed fruit technology arose, which was the induction of reciprocal translocation strains by X-ray irradiation to breed diploid, heterozygous less seed watermelon^18,19^. The typical cytogenetic characteristics of reciprocal translocation is one complex pair (such as a ring or chain) of several chromosomes at meiosis, causing semisterile gametes to produce fewer or no seeds. Pollen semisterile plants may have been heterozygous for translocation, and pollen-fertile plants may have been homozygous for translocation or have no translocation. At present, with the increasing number of molecular breeding approaches for facilitating crop improvement to acquire desirable traits, it would be possible to acquire less seed watermelon by molecular breeding methods. For example, clustered regularly interspaced short palindromic repeats (CRISPR)-associated protein (Cas) technology has been applied in plant breeding to improve single or multiple traits^20–22^. Recently, with the emergence of CRISPR-Cas9-mediated induction of heritable chromosomal translocations^23^, it has become possible for watermelon breeders to create translocation strains of less seed watermelon using this technology. However, the essential chromosome size or range for inducing reciprocal translocations between heterologous chromosomes remains unknown. Therefore, studying the phenotype of translocation plants and further locating the translocation segment is valuable for future translocation-related less seed watermelon breeding.

In the present study, we characterize a novel spontaneous chromosomal translocation line, “148”, which exhibits a less seed phenotype in its F_1_ hybrid, but sets normally when selfed. We further found through whole-genome resequencing and genetic mapping analysis that a translocated 2.09 Mb region on chromosome 6 caused defects during meiosis, which further led to diploid less seed watermelon. Our study reports a new promising method for breeding elite less seed watermelon at the diploid level as well as a fragment size reference for less seed watermelon breeding through artificially induced chromosome translocation.

## Results

### The fruits of the “148 × JM” F_1_ hybrid line displayed a less seed watermelon phenotype

**Fig. 1:**
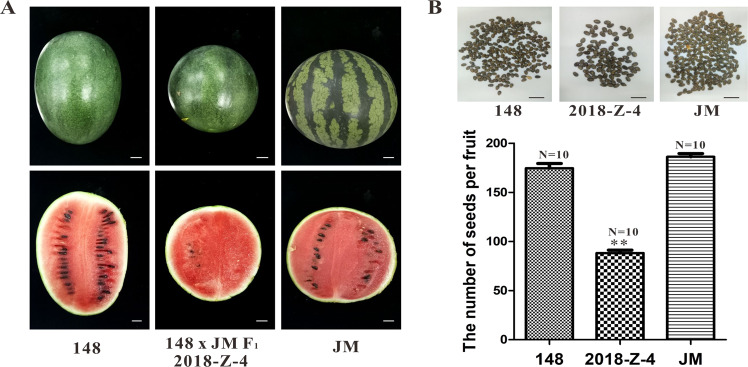
Phenotypic characterization and statistical analysis of less seed watermelon line “148 × JM” F_1_ hybrid, normal female parent line “148”, and male parent line “JM”. **A** Fruit morphology and longitudinal sections of three watermelon lines. Bars=2 cm. **B** The seed number per fruit of the three watermelon lines. *N* = 10 represents ten fruits for each line. Values are the means + SD. The statistical data were analyzed with a one-tailed Student’s *t* test to evaluate significance. Double asterisks indicate significant differences with respect to the watermelon parent lines (*t* test at *p* < 0.01)

To study fruit and seed development in a large-scale plantation we identified a natural spontaneous watermelon line, “148”, which exhibits a less seed phenotype in its F_1_ hybrid, but sets seeds normally when selfed. To further ascertain the details of the “148” mutant phenotype, the seed number of self-crossing seeds among the female “148” parent, the male parent “JM” and their F_1_ hybrid “2018-z-4” were comparatively analyzed. Intuitive observations of the cross-sections of fruits showed that the seed number of “2018-z-4” watermelon fruit was significantly less than that of either the female parent “148” or the male parent “JM” (Fig. 1A). Ten fruits were harvested from each watermelon source, and the seed number was analyzed statistically. The average seed number per fruit from “148” and “JM” were 175 and 186 grains, respectively, which were not significantly different. However, the average seed number of the F_1_ hybrid “2018-z-4” was only 88 grains per fruit, which was 50.3% and 47.3% of that of the two parental watermelon lines, respectively, indicating remarkably fewer seeds in “2018-z-4” watermelon than those of its parent lines (Fig. 1B). Fruit is considered to be less seed if it produces no seeds, traces of abortion seeds, or a significantly reduced number of seeds^12^. Taken together, these results demonstrate that the F_1_ hybrid “2018-z-4” displayed less seed phenotypic characterization, whereas the parental watermelon fruits possessed normal amounts of seeds.

### Less seed phenotype was caused by gamete semisterility in the “2018-z-4” watermelon line

**Fig. 2:**
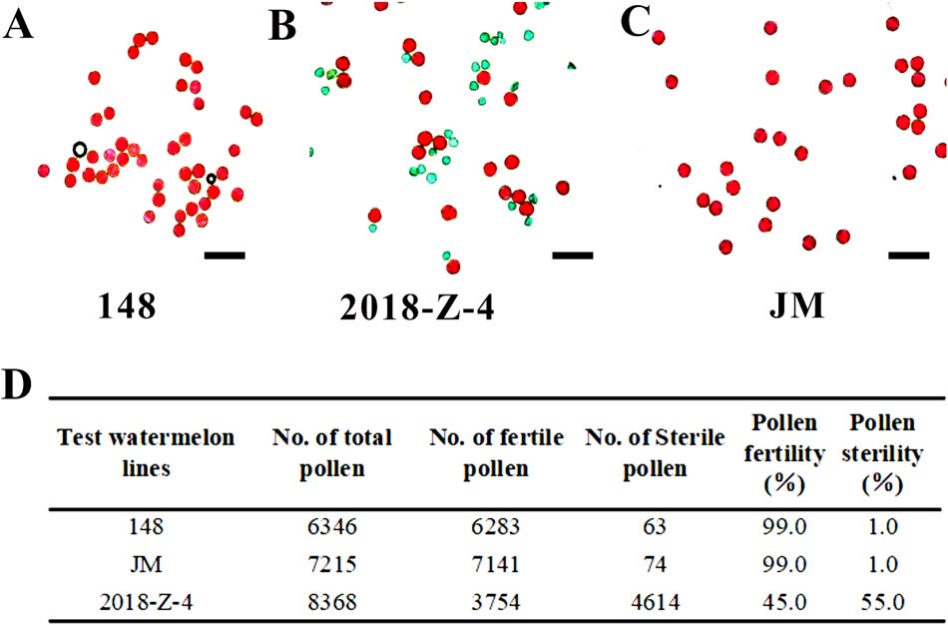
Pollen fertility analysis among watermelon lines “148”, “JM” and “2018-Z-4”. **A** The normal pollen grains of the female parent “148” line with Alexander’s stain. **B** The semisterile pollen grains of the F_1_ hybrid. **C** The normal pollen grains of the male parent “JM” line with Alexander’s stain. **D** The pollen fertility statistical analysis among “148”, “2018-Z-4” and “JM lines”. Scale bars = 10 μm in **A**, **B**, and **C**

Male and female sterility is one of the main causes of less seed fruit production^24–27^. Male and female gamete development is highly synchronized and almost identical in plants. Due to the difficult accessibility of female gametophytes, which are deeply embedded in sporophytic tissue, pollen viability was evaluated by Alexander’s staining method. Fresh pollen grains from ten male flowers of three watermelon lines at the time of another dehiscence were stained with Alexander’s solution^28^. Viable or fertile pollen grains exhibited green cell walls and red cytoplasm after staining. Nevertheless, nonviable or sterile pollen grains with shriveled shapes stained green due to a lack of cellular content. Alexander’s staining results suggested that nearly all pollen grains of the “148 and JM” parent lines were viable with round and plump shapes and showed red-stained cytoplasm (Fig. 2A, C). A portion of pollen grains from the F_1_ hybrid “2018-Z-4” were viable, but the others were sterile (Fig. 2B). The pollen grains of ten flowers from each watermelon line were obtained, and ten stained preparations were made. Respectively, 147, 235, and 228 microscope fields were captured for statistical analysis, and 6346, 7215, and 8368 pollen grains from “148”, “JM” and “2018-Z-4” watermelon lines were scored at random. The results demonstrated that pollen fertilities of “148 and JM” parent lines were as high as 99%. However, consistent with the seed number reduction in the F_1_ hybrid line, the pollen fertility of the F_1_ hybrid “2018-Z-4” was 55%, representing a pollen semisterility phenotype characteristic of hybrids.

**Fig. 3:**
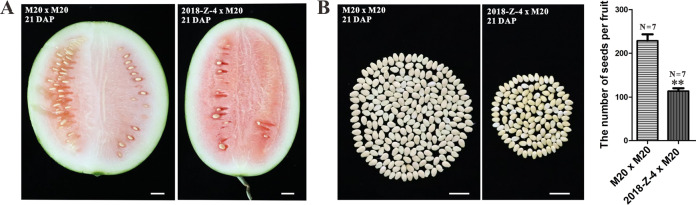
Comparison of seed numbers between the “M20 × M20” and “2018-Z-4 × M20” cross-combinations. **A** Longitudinal sections of fruit from watermelon sources. Bars = 2 cm. **B** The seed number per fruit of two watermelon lines. Bars=2 cm. *N* = 7 represents seven fruits for each line. Values are the means + SD. The statistical data were analyzed with a one-tailed Student’s *t* test to evaluate significance. Double asterisks indicate significant differences with respect to the watermelon parent lines (*t* test at *p* < 0.01). DAP stands for days after pollination

However, in most higher plants, the number of pollen grains landed on the stigma is far greater than the number of ovules; therefore, a decrease in pollen fertility may not result in such a dramatic reduction in seed number production. We suspected that seedlessness is most likely caused by defects in megaspore or ovule development. However, the investigation of female gametophytes is technically challenging because female gametes are embedded deeply inside ovarian tissue. However, we were able to determine whether ovule development or female gamete development were defective by crossing “2018-Z-4” as a female parent with a watermelon variety with normal pollen development. Therefore, we chose “M20” as the pollen donor, a diploid watermelon material that sets seeds normally. Results indicated that the seed number in the “2018-Z-4 × M20” cross-combination was reduced by 50% compared to the control “M20 × M20” (Fig. 3). Considering the synchronized development of male and female reproductive processes, the meiosis abnormality of microspore mother cells that we have identified should be considered a meiosis abnormality problem of spore mother cells as well. Additionally, our cross-pollinating results showed that the seed number reduction in the F_1_ hybrid “2018-z-4” watermelon line was caused by female gamete semisterility.

### Pollen semisterility was caused by the reciprocal translocation of chromosomes during meiosis in the “2018-z-4” F_1_ hybrids

Meiosis occurring in pollen mother cells is a fundamental process that ensures fertility^29,30^. Meiosis comprises one round of DNA replication, followed by two rounds of chromosome segregation, designated as meiosis I (homologous chromosomes segregate in the first division) and meiosis II (sister chromatids segregate in the second division). Meiosis I mainly includes leptotene, pachytene, diakinesis, prometaphase I, metaphase I, anaphase I, and telophase I. Meiosis II consists of prophase II, metaphase II, anaphase II, and telophase II.

**Fig. 4:**
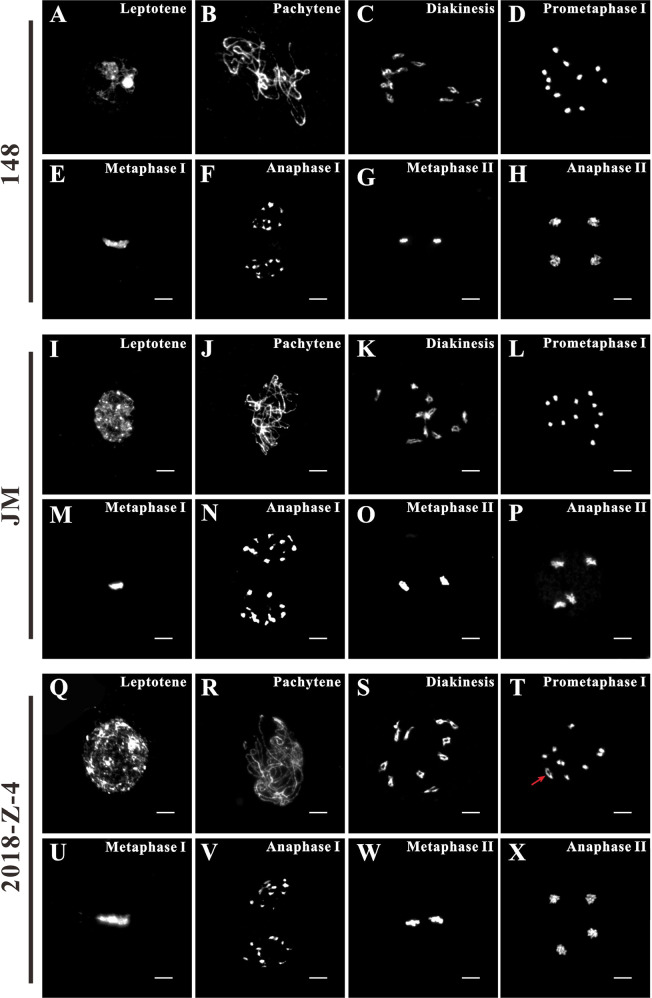
Aberrant chromosome behavior of reciprocal translocation in early prometaphase I during meiosis in the “2018-z-4” line compared with those in “148” and “JM” lines. Normal meiosis from leptotene to anaphase II in three watermelon lines. **A**, **I**, and **Q** Leptotene, chromosomes are shown as thin threads. **B**, **J**, and **R**. pachytene, fully synapsed chromosomes appear as thick threads. **C**, **K**, and **S** diakinesis, chromosomes are moderately condensed as bivalents. **D**, **L** Prometaphase I, chromosomes remain compacted. **E,** **M**, and **U** Metaphase I, the bivalents were aligned in an orderly fashion at the equatorial plate. **F**, **N**, and **V** Anaphase I, the homologous chromosomes separated and moved toward opposite poles. **G**, **O**, and **W** Metaphase II, two groups of condensed chromosomes are arranged at the equatorial plate. **H**, **P**, and **X** anaphase II, chromatids separated from each other and moved toward opposite poles of the tetrad. Abnormal early prometaphase I during meiosis in the “2018-z-4” line**. T** The pollen-semisterile plant line “2018-z-4” showed nice bivalents and a ring of four chromosomes at prometaphase I during meiosis. That was ⊙4 + 9_II_ at prometaphase I of F_1_ hybrids with pollen semisterility. The red arrow points to the position of the tetravalent ring. The images were obtained from DAPI-stained chromosome spreads following the fixation of floral buds. Scale bars=10 μm. ⊙4 + 9_II_ indicated a round-shaped complex pair of a quadrivalent and 9 bivalents

During meiosis, abnormal chromosomal behavior, including heterozygosity in the rearrangement of chromosomal segments, also termed “structural hybrids”, may result from interchanges, reciprocal translocations, or translocations, has been confirmed to be one of the major causes of gamete sterility in plants^31^. In addition, pollen semisterility is a good index of the occurrence of reciprocal translocation^18^. In order to further identify and verify the cause of pollen semisterility in the “2018-z-4” line, chromosome behavior during meiosis in pollen mother cells in parent and F_1_ hybrid sources was carefully assessed. After enzymatic hydrolysis, meiocytes were released from the anthers and were counterstained with DAPI (4’, 6-diamidino-2-phenylindole) for chromosome behavior analysis. The cytological observation revealed that the chromosomal behavior of parental lines was normal with regard to the progression of meiosis (Fig. 4A–P). Before prometaphase I, the meiotic chromosomal behavior in the F_1_ hybrid “2018-z-4” was indistinguishable from that of its parental lines. At prometaphase I, 11 condensed bivalents were clearly counted in the plants with normal pollen fertility (Fig. 4D,L). However, an obvious defect was identified at prometaphase I. The meiocytes in hybrid “2018-z-4” had nine bivalents and a ring of four chromosomes (Fig. 4T), indicating the occurrence of reciprocal translocation^18^. The frequency of a complex pair of four chromosomes with nine bivalents was approximately 19%, or 25 out of 127 microspore mother cells. The lower-than-expected ratio of the complex pair might be a result of sample preparation. Results revealed that pollen semisterility in the hybrid was caused by the reciprocal translocation of chromosomes.

**Fig. 5:**
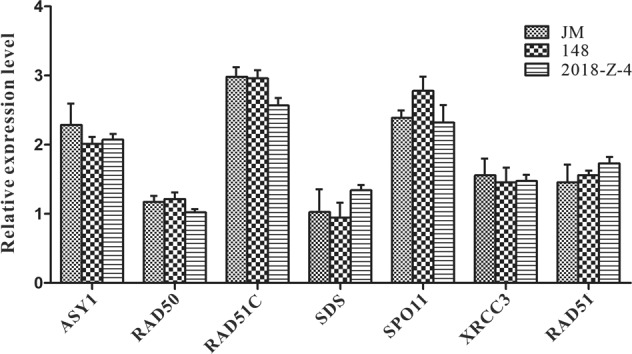
Relative expression levels of chromosome pairing and synapsis genes during meiosis in “JM”, “148” and F_1_ hybrid “2018-Z-4” watermelon lines. RT-qPCR analysis showed the relative expression levels of seven genes that specifically function in homologous pairing and synapsis during meiosis in parental watermelon lines and the F_1_ hybrid “2018-Z-4” line. All experiments were performed with three independent replicates. The *ClACTIN* gene (gene ID: *Cla007792*) was used for the normalization of RT-qPCR results

Moreover, to further determine whether pollen semisterility was caused by reciprocal translocation of chromosomes instead of gene expression, we performed RT-qPCR (reverse transcription-quantitative PCR) to compare the expression levels of seven critical meiotic genes in parental watermelon lines and F_1_ hybrid plants; the seven genes were *ASY1*^32^, *RAD50*^33^, *RAD51*^34^, *RAD51C*^35^, *SDS*^36^, *SPO11*^37^, and *XRCC3*^38^ (Supplementary Table S1). These genes play essential roles in meiosis. ASY1 localizes to axis-associated chromatin and is required for meiotic chromosome synapsis in *Arabidopsis* and *Brassica*. *RAD50* function is essential for telomere maintenance in *Arabidopsis*. The *RAD51* gene is required for normal meiotic chromosome synapsis and double-stranded break repair in *Arabidopsis*. *Arabidopsis* RAD51, RAD51C, and XRCC3 proteins form a complex and facilitate RAD51 localization on chromosomes for meiotic recombination. *SDS* (*SOLO DANCERS* gene) encodes a cyclin-like gene and is required for homolog interaction during meiotic prophase I in *Arabidopsis*. *AtSPO11-1* is necessary for efficient meiotic recombination in plants. RT-qPCR revealed that there was no significant difference among the relative transcript levels of the seven genes from the “JM”, ‘148” or F_1_ hybrid “2018-Z-4” watermelon lines (Fig. 5). These data indicate that the normal function of the seven genes did not account for the pollen semisterility phenotype of the F_1_ hybrid “2018-Z-4”. Collectively, all of the results strongly demonstrated that the gamete semisterility phenotype was caused by the occurrence of a reciprocal translocation of chromosomes during meiosis in the F1 hybrids.

### The less seed phenotype was caused by one chromosomal segment translocation of

To further investigate the translocation of the chromosomal segment and explore the less seed fruit mechanism, an F_2_ segregation population was generated by self-pollinating “2018-Z-4” F_1_ hybrid plants, and the seeds were harvested. We first examined pollen viability with Alexander’s stain. The results suggested that the pollen from the F_2_ population was segregated into two phenotypes: normal and partially sterile. Table 1 shows that there were 25 fertile and 27 semisterile plants among all F_2_ segregation plants (52 individuals in total), exhibiting a 1:1 Mendelian segregation ratio. In addition, the pollen viability of fertile plants in the F_2_ population was consistent with that of the parents (Table 1, Fig. S1). As described above, a single segment reciprocal chromosomal translocation produced the genetic behavior of gamete semisterility in the proceeding generation. These results suggest that pollen the semisterile plants had been heterozygous for translocation and that the pollen fertile plants had been homozygous for the translocation or had no translocation. This further confirmed that the less seed phenotype of the F_1_ hybrid plants was caused by gamete semisterility, which was controlled by a single segment reciprocal chromosomal translocation.

**Table 1:** The pollen fertility statistical analysis in F_2_ segregation plants

No. F_2_ plant individuals	No. of total pollen	No. of fertile pollen	No. of sterile pollen	Pollen fertility (%)	Pollen Sterility (%)
25	9218	9075	143	98.4	1.6
27	10754	4946	5808	45.9	54

### Genome-wide identification of high-confidence SNPs and indels for linkage mapping to the translocation fragment

To obtain enough SNPs (single-nucleotide polymorphisms) and indels (insertions and deletions) for developing polymorphic markers and localizing the chromosome translocation segment, genomic DNA of two parental lines, “148” and “JM”, was extracted and resequenced. After removing the low-quality reads, we obtained a total of approximately 7.93 and 49.79 GB of clean data from “148” and from “JM”, with 90.82% and 91.85% Q30 percentage values, respectively (Table 2). Next, 99.53% and 99.58% of these clean reads for “148” and “JM” were successfully mapped onto the watermelon reference genome, which enabled us to develop a total of 540,269 SNPs and 195,378 indels between two parental line genomes. After removing the low-quality sites with reading counts <20 and quality <40, 169,973 high-confidence SNPs and 5261 indels were successfully obtained and used to develop CAPS markers in the next mapping strategy (Supplementary Table S2).

**Table 2:** Detailed characteristics of DNA-seq data of the parental lines “148” and “JM”

	148	JM
Clean data (GB)	7.93	49.79
Q30 percentage	90.82%	91.85%
GC%	34.12%	34.92%
Mapped reads	99.53%	99.58%
Coverage ratio	98.10%	98.22%

### A 2.09 Mb chromosome translocation on chromosome 6 was responsible for the less seed phenotype based on mapping cloning

**Fig. 6:**
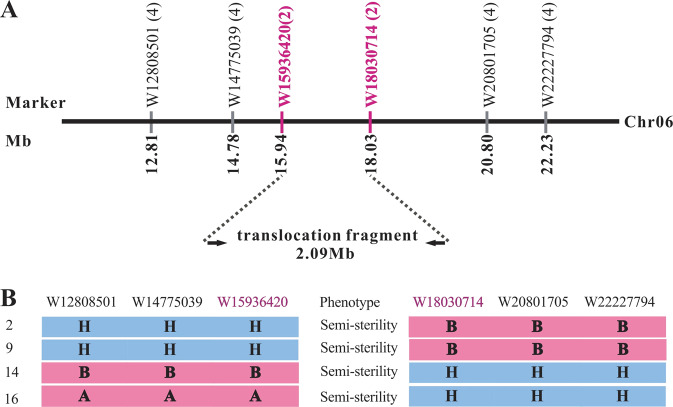
Map-based genetic location of the translocation fragment in watermelon A. Mapping translocation fragment using 52 F_2_ segregation population individuals. The translocation fragment was delimited to the region between markers W1593642 and W18030714 on chromosome 6. The numbers in brackets after each marker indicate the number of recombinants between markers and phenotypes. **B**. Marker genotypes of the recombinants near the translocation fragment between markers W1593642 and W18030714. The alleles are marked according to their origin: A., pollen-fertile homozygote (AA); B., pollen-fertile homozygote (aa); and H., pollen semisterile heterozygote

To map the chromosome translocation segment that caused the less seed phenotype and further provide a chromosome reference size for artificially inducing heritable chromosomal translocations in the plants, polymorphic markers were designed on the 11 chromosomes and then used to genotype individuals in the F_2_ segregated plant population described above. The results showed that the marker W14775039 on chromosome 6 was linked with the less seed phenotype (Fig. 6A). To further verify the location of the translocation segment, other polymorphic markers, W12808501 (on the left side of W14775039), W15936420, W18030714, W20801705, and W22227794 (on the right side of W14775039), were designed to screen this F_2_ segregation population. Subsequent linkage analysis demonstrated that markers W12808501, W14775039, and W15936420 were located on the same side of the translocation segment and each had four recombinants at distances of 12.81, 14.78, and 15.94 cM, respectively. Moreover, markers W18030714, W20801705, and W22227794 were situated on the other side, with two, four, and four recombinants at 18.03, 20.80, and 22.23 cM genetic distances from the translocation fragment location, respectively (Fig. 6B). The linkage analysis results revealed a translocation fragment locus narrowed down to an interval between markers W15936420 and W18030714, for two of the recombinants. Based on the “97103” watermelon reference genome, these two markers spanned 2.09 Mb. Therefore, these results confirmed that the 2.09 Mb chromosome translocation on chromosome 6 was responsible for the less seed phenotype.

## Discussion

Watermelon from the Cucurbitaceae family is an economically important vegetable crop for human consumption worldwide. According to the Food and Agricultural Organization of the United Nations (FAO, http://faostat.fao.org), the global area of watermelon production accounts for 7% of global vegetable production^39^. The annual production quantity is 118.4 million tons (MT), and the production value is US $ 33.9 million (FAO, 2017). Meanwhile, watermelon is one of the most extensively consumed fruit crops in the world and is among the top five most consumed fresh fruits (http://faostat.fao.org). Watermelon fruits include seeded (i.e., normal diploid) and hybrid less seed (i.e., triploid) types. less seed watermelon fruits have become more popular and desirable throughout the world due to easy consumption, gustatory advantages, consumer preference, and greater economic value to growers over traditional seeded cultivars^7,40,41^. For example, in the United States, the popularity of less seed watermelon has steadily increased over the past decade^42,43^. Less seed watermelon accounts for over 80% of watermelon production (AgMRC, 2018).

Because the current production methods of triploid less seed watermelon are associated with shortcomings, it is necessary to develop a new method to produce less seed watermelon fruits for watermelon breeders. Pursuant to this aim, watermelon researchers are continuously investigating and trying to discover diploid less seed watermelon lines and explore their formation mechanisms. However, few diploid less seed watermelons have been identified, and the cytological and molecular mechanisms remain largely unknown. Our findings here demonstrate that we have successfully obtained a diploid hybrid less seed watermelon variety, caused by a 2.09 Mb fragment translocation on chromosome 6, which induces abnormal metaphase I during meiosis.

### A 2.09 Mb fragment translocation on chromosome 6 caused less seed watermelon fruits

In the process of watermelon selection and breeding research in our present study, we identified a watermelon line, “148”, which exhibits normal growth and development. There was no significant difference between the seed number from “148” and that of the control (Fig. 1). However, F_1_ hybrids generated sharply decreased seed numbers (approximately 50% less) compared to the parents when crossed with the male parental watermelon line “JM”. In addition, there was no significant difference in plant growth development except for the less seed phenotype of “148” watermelons, including leaf number, plant type, and growth rate.

In eudicots, there are three major processes for plant sexual reproduction: spore mother cell differentiation; meiosis; and continuous mitosis^44^. Theoretically, knocking out critical genes that play roles in plant sexual reproduction will produce diploid less seed watermelon. However, the obstacle is how to obtain seeds during breeding. However, for creating less seed watermelon by the chromosome translocation method, there are no gene disruptions, and plants will be fertile, if the translocation is homozygous. A less seed phenotype will only appear in the F_1_ generation once the homozygous translocation parent crosses with other watermelon lines.

In general, female gamete development has a direct impact on seed formation and development. However, female gametes, the egg cells of higher plants, are embedded deep inside the ovary, which presents many difficulties for investigating and examining female or egg cell fertility. However, male gamete sterility (MS) and female sterility are synchronous in nature, especially when defects occur during meiosis^25,45–47^. Therefore, in our work, we carefully examined the male gamete or pollen viability of parental and F_1_ hybrid plants. Pollen viability analysis showed that the pollen fertility of parental watermelon lines was normal, but F_1_ hybrid plants exhibited pollen semisterility defects.

To explain the cause of semisterility in the F_1_ hybrid, we proposed the following model: In the F_1_ hybrid line “2018-Z-4” at meiotic metaphase I, reciprocal translocation occurred between two nonhomologous chromosomes, resulting in the formation of a complex pair of a quadrivalent ring. Then, at anaphase II, this quadripartite group of translocation chromosomes is separated by two patterns of segregation: alternate and adjacent segregations. In the case of alternate segregation, the translocation chromosomes segregate to one pole, and the normal homologs pass to the other, thus yielding normal and balanced gametes. In the case of adjacent segregation, there are two types: adjacent-1, in which the two chromosomes with homologous centromeres move to opposite poles, and adjacent-2, in which they migrate to the same pole. In adjacent segregation, there is nondisjunction for certain chromosome segments. Therefore, only alternate segregation results in combinations that are not deficient, whereas adjacent-1 and adjacent-2 segregation produce unbalanced or deficient gametes^48^. Deficient spores abort and are unable to complete their divisions or the development necessary to form the gametophyte. Ultimately, plants from the F_1_ hybrid line “2018-Z-4” produced an approximately 1:1 ratio of aborted pollen and normal pollen (i.e., pollen semisterility) (Fig. S2). In addition, for megaspore and microspore mother cells, the basic process of meiosis shares high similarities. However, megaspore mother cells or female gametes of watermelon are inaccessible for cytological chromosome observation. Therefore, in our work, we could only infer female gamete viability by using the pollen viability of parent and F_1_ hybrid plants. However, when “2018-z-4” crossed a male parent with normal pollen, the seed number also reduced to 50% of that of the parental line. This confirms that less seed phenomena arise from defects of female gametes. Meanwhile, the less seed phenotype did not occur solely by chance in the crossing combination of “148 × JM”. When “148” was crossed with other watermelon lines, such as “148 × Y83”, “148 × JF” and “M08 × 148”, all F_1_ hybrid lines showed a less seed phenotype (Fig. S3), which further supports that “148” watermelon is a homologous translocation material and that F_1_ hybrid plants produced less seed fruits.

Chromosome pairing and segregation during meiosis were regular (bivalent) in parent species, but F_1_ hybrids were found to be altered abnormally with the formation of one quadrivalent ring of four chromosomes at prometaphase I during meiosis. The formation of a quadrivalent ring is a typical characteristic of chromosomal reciprocal translocation^18^. The subsequent reciprocal chromosomal translocation occurred by two types of segregation, alternate or adjacent segregation, at anaphase II, and resulted in unbalanced gametes with a missing chromosome segment in the F_1_ hybrids^49^. Then, the gametes with incomplete chromosome complements in the hybrids contributed to their loss of fertility. In brief, we explored the cause of less seed watermelon fruits from a cytogenetic perspective and laid the groundwork for future molecular biological studies.

**Fig. 7:**
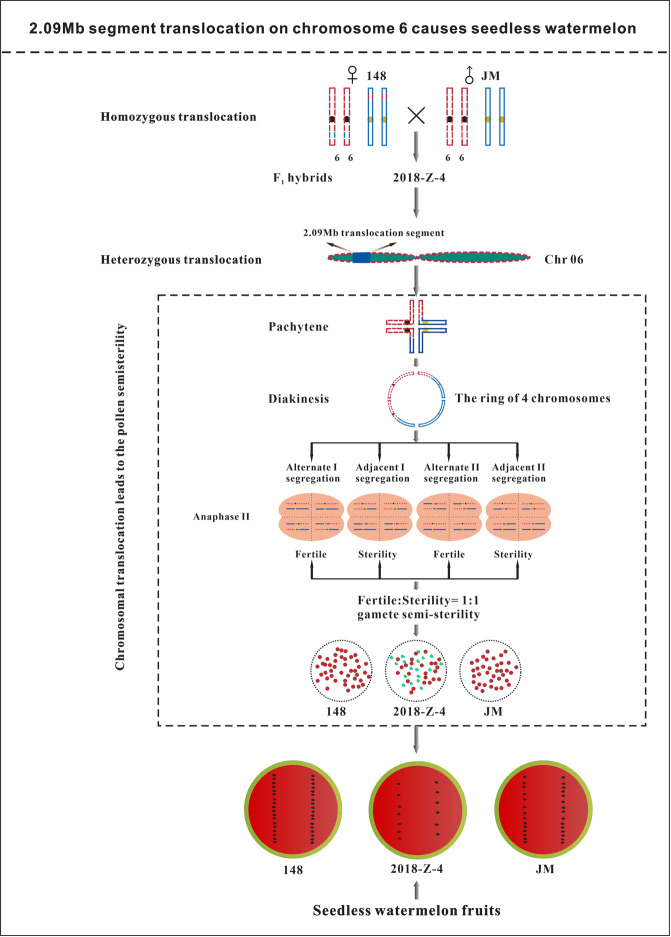
Proposed model for less seed phenotype caused by 2.09 Mb fragment translocation on chromosome 6. The homozygous translocation watermelon line “148” crossed with a normal watermelon line “JM” produced F_1_ hybrids “2018-Z-4”, where were heterozygous for the chromosome translocation. A 2.09 Mb fragment translocation on chromosome 6 caused abnormality during meiosis and produced “semisterility” which led to less seed watermelon in F_1_ hybrids

The question arose as to which chromosome and what size of chromosomal translocation fragment caused the gamete semisterility and led to the less seed phenotype. Chromosomal translocation is defined as a genome abnormality in which a chromosome breaks and either the whole chromosome, or a portion thereof, reattaches to a different chromosome^50^. Structural chromosome abnormalities have been reported to play important roles in the utilization of alien genes and chromosome ploidy manipulation^51–53^ and in understanding chromosome-level genomics and biology^54–56^. Chromosome translocation was first found in plants in 1901 by Hugo de Vries, and there are many methods for detecting the cytogenesis of affected cells, including DNA resequencing to obtain isozyme markers, as well as FISH and GISH (fluorescence genomic in situ hybridization)^57,58^. Linkage analysis indicated that the translocation fragment leading to the less seed phenotype was delimited to a 2.09 Mb genomic region. We further propose a model for the less seed watermelon phenotype and mechanism in meiosis during seed development (Fig. 7). However, the minimum length required for the chromosome translocation to generate less seed watermelon needs to be confirmed.

### Future perspectives

Spontaneous reciprocal translocation has been identified in some important economic food crops. Reciprocal translocation has been reported in species, such as barley, bean, wheat, and maize^51,59–65^. Reciprocal translocation in horticultural crop species has mainly been identified and investigated in edible banana cultivars^66^. However, spontaneous reciprocal translocation has rarely been identified in watermelon cultivars^67^. Previously, in 1967, induced chromosome translocation in watermelon lines were generated by irradiating watermelon seeds with X-rays; some lines of reciprocal translocation homozygotes were found in the progenies. For molecular breeding strategies, CRISPR/Cas9 genome editing technology has been applied in plant breeding to improve single or multiple traits^68–71^. Furthermore, in the model plant Arabidopsis, CRISPR–Cas9-mediated technology has been successfully used to restructure plant chromosomes and induce reciprocal translocation between heterologous chromosomes^72^. In the food crop maize, the application of CRISPR–Cas9-mediated technology resulted in 75.5-Mb pericentric inversion in chromosome 2, providing opportunities for the development of new maize varieties with improved traits^73^. CRISPR-Cas9 genome editing technology has great potential for restructuring watermelon genomes to produce less seed watermelon.

## Materials and methods

### Plant sources and growth conditions

Two watermelon lines “148” and “JM” were used as parents, and their F_1_ hybrid plants (148 × JM F_1_ generations), F_2_ segregation population plants M20, Y83, JF, M08, and wild-type watermelon ZTC in this study were grown in a plastic greenhouse at Northwest A&F University, Yangling, China under natural conditions. “148” is a conventional watermelon germplasm material and East Asian Ecotype (Henan conventional species). “JM” is the homozygous germplasm of watermelon from Japan and the female parent of “Jingxin”. Both “148” and “JM” watermelon sources are diploid and pure watermelon lines.

### Seed number statistic

The three watermelon sources, “148”, “JM”, and “2018-Z-4”, were self-pollinated individually and fruits were then separately harvested. The harvested fruits were examined for morphology differences and cut longitudinally to count the number of seeds in the longitudinal section.

### Pollen fertility examination

The pollen viability examination was performed as previously reported^74^. Fresh pollen grains were collected and stained with Alexander’s stain. The specimens were mounted, covered with coverslips, and examined microscopically. The aborted pollen stained green, and the nonabortedpollen stained red to deep red. The numbers of fertile and sterile pollen grains were counted under the microscope. Each specimen was examined three times, and the pollen from three fields of view per slide was counted.

### Observation of chromosome behavior in meiosis

To visualize meiotic chromosome behavior and determine phenotype, a procedure called chromosome spreading is used to partially separate the chromosomes after limited digestion of the meiocytes to loosen the cell walls. In our study, we performed this procedure as previously reported^75^. The flower buds were fixed in Carnoy’s fixative in ethanol-acetic acid (3:1) at room temperature for at least 12 h. The fixed buds were then rinsed three times with citrate buffer (10 mM sodium citrate, pH 4.5). Next, the buds were digested in an enzyme solution consisting of 0.3% cytohelicase, 0.3% pectolyase, 0.3% cellulase, and 1.4% β-glucuronidase in citrate buffer at 37 °C for 3 h. Thirteen microliters of 60% acetic acid was added to the enzyme-digested bud to clear the materials and covered with a coverslip to press it down. The specimens were next placed in the freezer at −80 °C. The slides were dried at room temperature and stained with DAPI. Finally, DAPI-stained cells were viewed under a fluorescence light microscope (OLYMPUS BX 63).

### RT-qPCR

To examine the expression levels of seven genes that specifically function in homologous pairing and synapsis during meiosis by RT-qPCR assay, the suitable flower buds from “148”, “JM” and “2018-Z-4” watermelon plants were selected by measuring their diameter to confirm that the plant development was in meiosis I; the diameters of the flower buds were between 1.0–2.5 mm. Total RNA was extracted from suitably sized flower buds from “148”, “JM”, and “2018-Z-4” watermelon lines using the RNeasy Plant Mini Kit. After DNase I digestion (Huayueyang Bio-Technology, Beijing, China), the reverse transcription reaction was performed with an M-MLV First-Strand Kit according to the manufacturer’s instructions. RT-qPCR was performed with SYBR Green and a Step One Plus Real-time system (ABI). The RT-qPCR amplification reaction was performed in a 20 μl volume containing 10.0 μl 2 × SYBR Green Mix (adding 0.2 μl Rox), 0.8 μl of forward and reverse primers, 0.8 μl of cDNA template (100 ng/μl) and 8.2 μl of ddH_2_O. The PCR amplification program included predenaturation for 3 min at 94 °C, followed by 40 cycles at 94 °C for 10 s and at 60 °C for 30 s. The specific primers were designed for RT-qPCR (Fig. 5). The comparative CT method (2^-ΔΔCT^ method) was used for the relative quantification of gene expression^76^, the watermelon *Actin* gene (gene ID: *Cla007792*) was used as an internal reference and PCR analysis for each gene was performed by three biological and three technical replicates. All primers are listed in Supplementary Table S3.

### Whole-genome resequencing of the two parental watermelon lines

The young leaves of two parental watermelon lines, “148” and “JM”, were selected for individual genomic DNA extraction using the CTAB method (cetyl trimethylammonium bromide). The extracted genomic DNA was digested with RNase I (Takara) to remove RNA. The quality of the DNA was examined on a 1% agarose gel, and the purity of DNA was measured by a Nanodrop 2000 spectrophotometer (Thermo Scientific, Wilmingto, DE). Using the BGISEQ 500 sequence platform, high-quality and pure genomic DNA was resequenced, generating 150 bp paired-end reads.

### Data analysis and marker development

The clean reads of the parental watermelon lines, “148” and “JM” were aligned to the “97103” watermelon reference genome through the use of BWA software with default parameters after removing the adaptors, reads with more than 10% unknown bases, and reads with more than 50% low-quality bases (*Q*-value ≤10). Raw SNP (single-nucleotide polymorphism) calling and indel calling were performed using GATK software. SNPs with a sequencing depth of <2 were filtered. SV sites (structure variation) were analyzed using BreakDancer software (http://breakdancer.sourceforge.net/), and CNV sites (copy number variation) were analyzed by SOAPcnv software. Next, high-confidence SNPs and indels were acquired and used to develop corresponding CAP markers (cleaved amplified polymorphic sequences) with Geneious software (http://www.geneious.com). Genomic DNA was extracted from young leaves using the CTAB method and used as templates for further PCR amplification. All PCR products were digested with corresponding restriction enzymes. After analyzing the enzyme-cut bands, polymorphic markers were directly used for the genetic mapping analysis. The sequencing data are accessible in the NCBI database under accession numbers SAMN19314832 and SAMN19314833. All the reads mapped on translocation fragments were visually investigated and compared between two parental lines using JBrowse software.

### Mapping of the chromosome translocation locus

To locate the chromosome translocation region, one polymorphic marker primer was designed for each chromosome based on the high-confidence SNPs identified previously. These 11 markers were used to screen an F_2_ segregation population with 52 individuals. These primers were used for PCR amplification based on the genomic DNA templates of F_2_ population plants exhibiting a pollen semisterility phenotype. The PCR products were digested with restriction enzymes and separated by 1% agarose gel electrophoresis. The linkage markers and the chromosome on which the translocation fragment was located were obtained based on enzyme-cut band analysis. After chromosome anchoring of the translocation segment locus, new flanking markers were developed to screen the F_2_ population. New flanking markers were used to screen the recombinants to narrow down the mapping interval. Primers of all the polymorphic markers are listed in Supplementary Table S4.

## Supplementary information

Supplemental Material

## Data Availability

The datasets generated during this study are available in the NCBI database (https://www.ncbi.nlm.nih.gov). The accession numbers are SAMN19314832 and SAMN19314833.
