# Supplementary material for: A 2.09 Mb fragment translocation on chromosome 6 causes abnormalities during meiosis and leads to less seed watermelon
Source: Hortic Res. 2021 Dec 1;8:256. doi: 10.1038/s41438-021-00687-9 (PMC8633341; doi:10.1038/s41438-021-00687-9)
Supplement: Supplementary file 1 — Supplemental Material [file 41438_2021_687_MOESM1_ESM.docx]

**A 2.09Mb fragment translocation on chromosome 6 causes abnormality during meiosis and leads to seedless watermelon**

**Shujuan Tian^#^, Jie Ge^#^, Gongli Ai, Jiao Jiang, Qiyan Liu, Xiner Chen, Man Liu, Jianqiang Yang, Xian Zhang, Li Yuan***

State Key Laboratory of Crop Stress Biology for Arid Areas, College of Horticulture, Northwest A&F University, Yangling, 712100, Shaanxi, China

**^#^** These authors contributed equally to this work.

* Correspondence: [lyuan@nwafu.edu.cn](mailto:lyuan@nwafu.edu.cn).

**Supplementary materials**

**
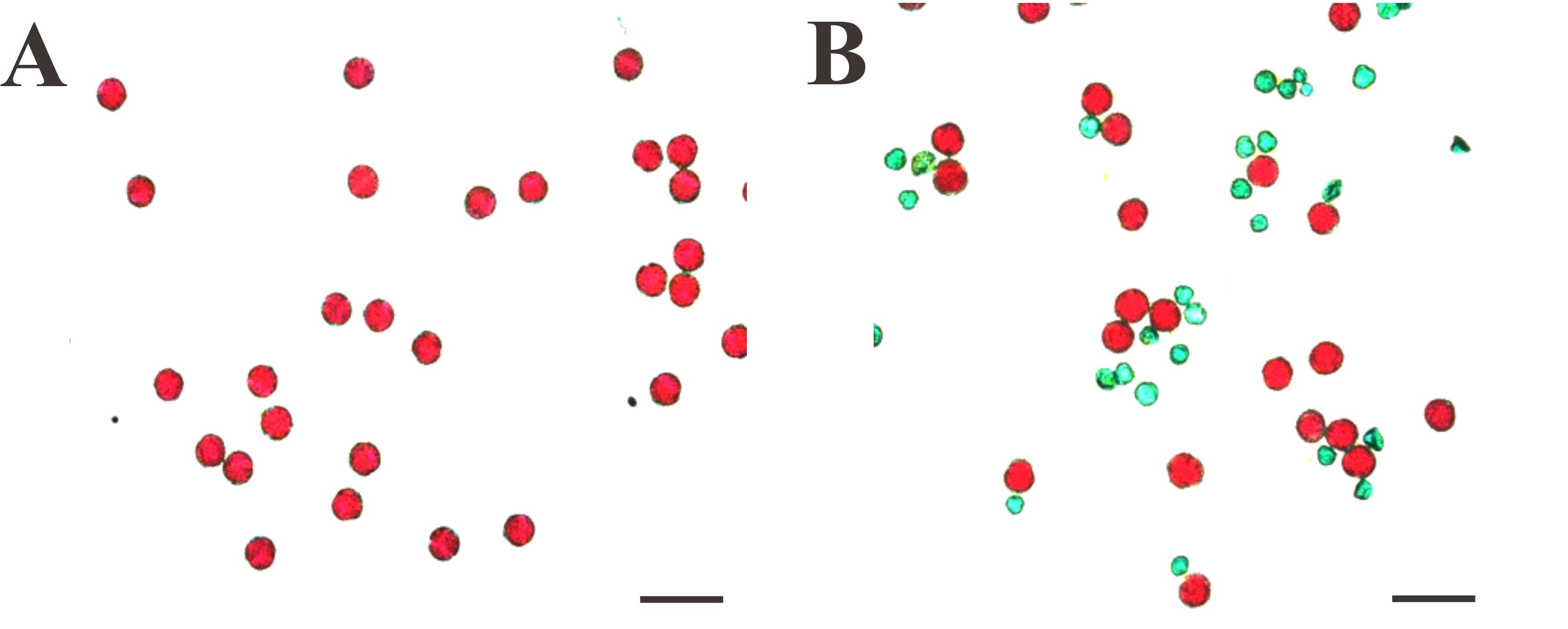
**

**Fig S1. The pollen fertility analysis in F_2_ segregation population plants. A**. The normal pollen fertility with Alexander’s stain. **B**. The semi-sterile pollen grains with Alexander’s stain. Scale bars=10μm.

**
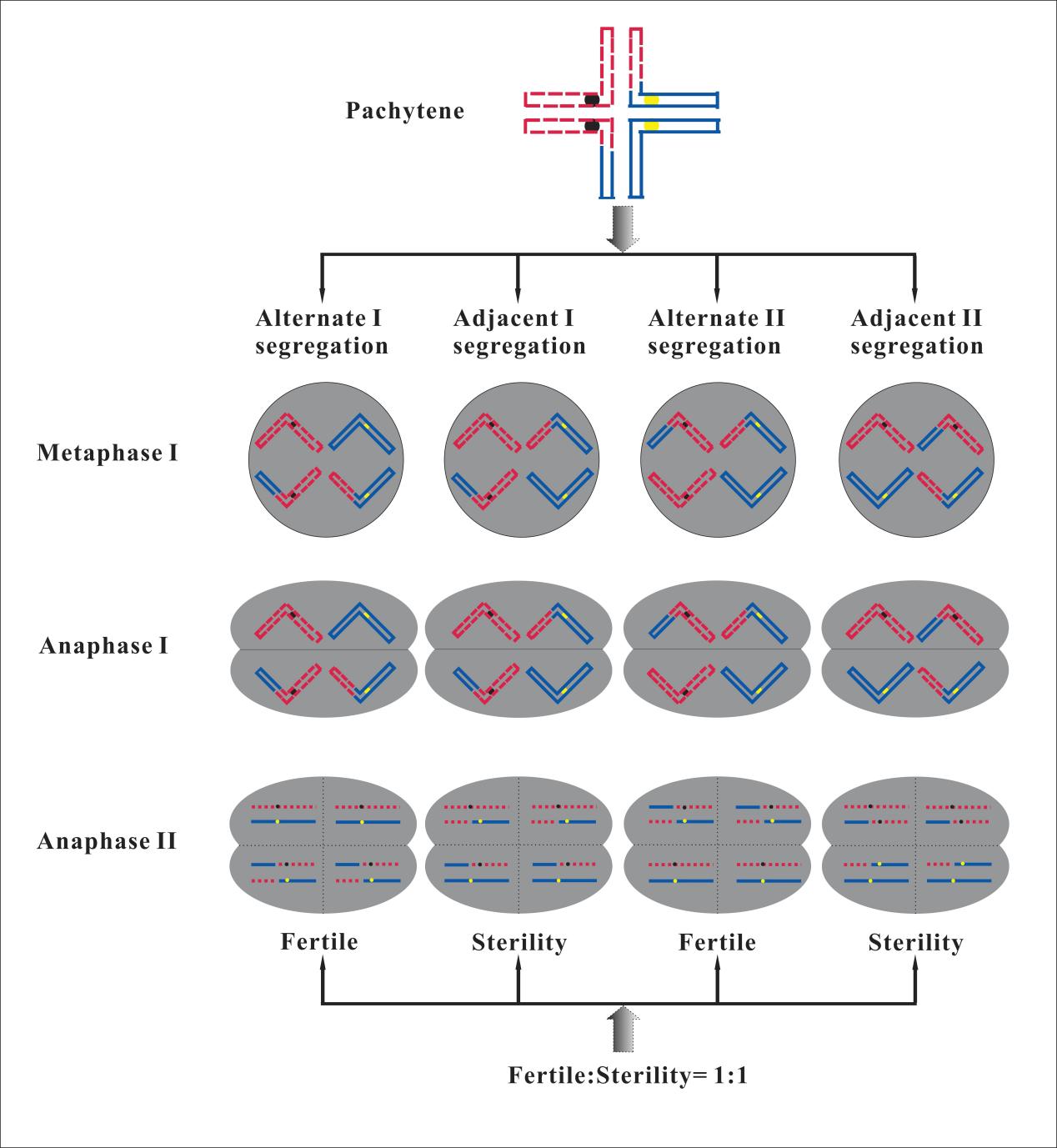
**

**Fig S2. Illustration of the pollen semi-sterility with the gametes produced by 2:2 segregation mechanisms of chromosomes involved in the reciprocal translocation.**


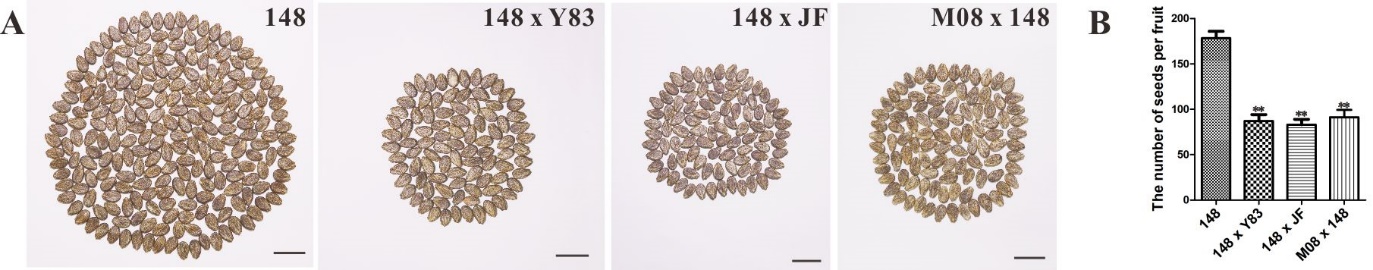


**Fig S3. The cross combinations including “148 x Y83”, “148 x JF”, “M08 x 148” produce the characterization of seedless fruits. A.** Seed number comparison of the 148 and cross combinations. **B.** The seed number analysis per fruit of the four watermelon lines. 8 fruits for each watermelon material were examined and analyzed. Values are the means+ SD. The statistical data were analyzed with one-tailed Student’s *t* test to evaluate the significance. Double asterisks indicate significance differences with respect to the watermelon parent lines (t test at p < 0.01).

**Table S1. Detailed information of 7 genes used in RT-qPCR that were related to homolog paring or synapsis during meiosis in watermelon.**

| **Gene ID** | **Homologous gene in Arabidopsis** | **Protein function** | **References** |
| --- | --- | --- | --- |
| *Cla022688* | *ASY1* | Homolog pairing | Armstrong et al., 2002 |
| *Cla021398* | *RAD50* | Homolog pairing, synapsis | Gallego et al., 2001 |
| *Cla011127* | *RAD51* | Homolog pairing, synapsis | Li et al., 2005 |
| *Cla021319* | *RAD51C* | Homolog pairing, synapsis | Abe et al., 2005 |
| *Cla022282* | *SDS* | Homolog pairing, synapsis | Azumi et al., 2002 |
| *Cla003301* | *SPO11* | Homolog pairing, synapsis | Grelon et al., 2001 |
| *Cla013251* | *XRCC3* | Homolog pairing, synapsis | Bleuyard and White, 2004 |

**Table S2. Chromosomal distribution of reliable SNPs and indels in watermelon.**

|  | **Chr1** | **Chr2** | **Chr3** | **Chr4** | **Chr5** | **Chr6** | **Chr7** | **Chr8** | **Chr9** | **Chr10** | **Chr11** |
| --- | --- | --- | --- | --- | --- | --- | --- | --- | --- | --- | --- |
| **SNPs** | 5838 | 6518 | 4638 | 4874 | 6156 | 11505 | 6833 | 3024 | 6519 | 7878 | 4830 |
| **Indels** | 3492 | 1138 | 1173 | 1127 | 1738 | 1998 | 1560 | 924 | 1690 | 1586 | 1227 |
| **Total** | 9330 | 7656 | 5811 | 6001 | 7894 | 13503 | 8393 | 3948 | 8209 | 9464 | 6057 |

**Table S3. The primer information of 7 genes used in RT-qPCR that were related to homolog paring or synapsis during meiosis in watermelon.**

| **Gene ID** | **Primer sequence (5'-3')** | **PCR product (bp)** | **Annealing temp (℃)** |
| --- | --- | --- | --- |
| *Cla022688* | F: AACAAGCTTGAAGGAGAGGCCA  R: TGATGTCACTCTCATGCGCGTT | 1368 | 60 |
| *Cla021398* | F: TGGTCCAAACGCCGAAAGTCTT  R: AAACATGCTGGTCGTCCTTCGT | 917 | 58 |
| *Cla011127* | F: TGCACAGGTAGATGGTTCAGCA  R: ATTTGGAACCGTGCTTCAGCCT | 675 | 58 |
| *Cla021319* | F: TTGCTCAGTGAAATGGCTCTTA  R: GCAGATTGAAGAGAGGGTGACT | 769 | 58 |
| *Cla022282* | F: AAAATGGAGGGTGAAGTTCTGA  R: AAGAACACCACTGATGCAGCTA | 541 | 58 |
| *Cla003301* | F: ACCAGAACAATGCCTCATCCCA  R: TGCAGCATTGAGTCCAACTCCA | 803 | 58 |
| *Cla007232* | F: TGGAGAAGCTAATGACCAGACG  R: GGTTCATTCCAGCAGCCAAATT | 924 | 58 |

**Table S4. List of all primers for 6 markers used in genetic mapping of translocation fragment.**

| **Marker name** | **Physical location** | **Primer sequence (5'-3')** | **PCR product (bp)** | **Endonuclease** | **Annealing temp (℃)** | **Marker type** |
| --- | --- | --- | --- | --- | --- | --- |
| W12808501 | Chr06:12808501 | F: GTTTGAAGTGAAAGCAACTCAA | 932 | *EcoR*I | 57 | CAPS |
|  |  | R: GGGGGCAATTGATTTCCATGG |  |  |  |  |
| W14775039 | Chr06:14775039 | F: GCCCTCAATTCGGTTTTCCC | 966 | *EcoR*V | 57 | CAPS |
|  |  | R: CCAACTGCGAAACCATGCAA |  |  |  |  |
| W15936420 | Chr06:15936420 | F: TGTGAAAAGTTTGGGGTCCT | 640 | *EcoR*I | 57 | CAPS |
|  |  | R: GGTAGCTGACGGTCTTTGCT |  |  |  |  |
| W18030714 | Chr06:18030714 | F: TCCCAACTGCTCTACTCCGA | 664 | *Msp*I | 57 | CAPS |
|  |  | R: TTGTGACGTGCGATCCTTCA |  |  |  |  |
| W20801705 | Chr06:20801705 | F: TTCGAAAGGCAGAGCAGAGG | 920 | *EcoR*V | 57 | CAPS |
|  |  | R: ACTGGTGCAGACAAGGATGG |  |  |  |  |
| W22227794 | Chr06:22227794 | F: AGACCAAGGGCTCATTTGGT | 940 | *Rsa*I | 57 | CAPS |
|  |  | R: TGTCGGCCCTTTCCATTCAA |  |  |  |  |
